# Nanodiamond-based nanostructures for coupling nitrogen-vacancy centres to metal nanoparticles and semiconductor quantum dots

**DOI:** 10.1038/ncomms11820

**Published:** 2016-06-08

**Authors:** Jianxiao Gong, Nat Steinsultz, Min Ouyang

**Affiliations:** 1 Department of Physics and Center for Nanophysics and Advanced Materials, University of Maryland, College Park, Maryland 20742, USA

## Abstract

The ability to control the interaction between nitrogen-vacancy centres in diamond and photonic and/or broadband plasmonic nanostructures is crucial for the development of solid-state quantum devices with optimum performance. However, existing methods typically employ top-down fabrication, which restrict scalable and feasible manipulation of nitrogen-vacancy centres. Here, we develop a general bottom-up approach to fabricate an emerging class of freestanding nanodiamond-based hybrid nanostructures with external functional units of either plasmonic nanoparticles or excitonic quantum dots. Precise control of the structural parameters (including size, composition, coverage and spacing of the external functional units) is achieved, representing a pre-requisite for exploring the underlying physics. Fine tuning of the emission characteristics through structural regulation is demonstrated by performing single-particle optical studies. This study opens a rich toolbox to tailor properties of quantum emitters, which can facilitate design guidelines for devices based on nitrogen-vacancy centres that use these freestanding hybrid nanostructures as building blocks.

## Abstract (web summary)

The coupling of nitrogen-vacancy centres with plasmonic and photonic nanostructures is usually studied by top-down preparation. Here, Gong *et al*. use a bottom-up approach to grow metallic and semiconducting nanoparticles on nanodiamonds, controlling the nanoparticle coverage, size and composition.

## Graphical abstract

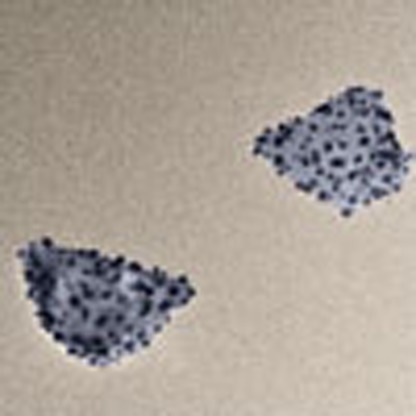

Nitrogen-vacancy (NV) centres in diamonds have attracted substantial interest over the past years as quantum emitters because of their use as bright, bio-compatible and photostable fluorescent emitters, as well as their unique spin characteristics that can be employed in quantum information processing and metrology^1^,^2^,^3^,^4^,^5^,^6^,^7^,^8^. The ability to control the interaction between such quantum emitters and photonic and/or broadband plasmonic nanostructures is crucial for the development of solid-state quantum devices with tunable performance. In prior studies, positioning NV centres close to plasmonic structures has been achieved by either top-down lithography or nanoscale manipulation, resulting in new physics^9^,^10^,^11^,^12^,^13^,^14^,^15^,^16^,^17^. Despite these promising developments, they have yet to materialize as freestanding structures, which is vital for realizing the full potential of NV centres in physical, biological and chemical applications.

When the diamond's size is reduced to nanometre scale (named ‘nanodiamond', ND), its confined NV centres are naturally close to the surface. This can therefore offer a unique opportunity to couple the NV quantum emitters in NDs to other external functional units on surface (for example, photonic, plasmonic or spintronic nanostructures), leading to the emergence of various physical interactions that can engineer unique characteristics of quantum emitters, depending on the interplay between their localized optical energy states^18^,^19^. For example, broadband-localized surface plasmons can typically lead to strong electromagnetic enhancement in the near field of metal nanoparticles, while Förster resonance energy transfer (FRET) might occur due to discrete energy level interactions (that is, long range dipole–dipole interactions) in the proximity of semiconducting nanostructures^20^,^21^,^22^,^23^. In addition, magnetic dipole coupling between the NV centres and the optically oriented spins in semiconducting or magnetic nanostructures on the surface might enable a new class of self-assembled quantum systems^24^,^25^,^26^. The strength of such fundamental interactions strongly depends on the inter-particle spacing and the nature of external functional units.

Thus far, most of the related work towards such fundamental couplings is limited to structures formed by either top-down lithography or individual particle manipulation, which is typically a very complicated and time consuming process that can be difficult to scale-up, limiting the scope of their applications^9^,^10^,^11^,^12^,^13^,^14^,^15^,^16^,^17^. Herein, we demonstrate a general facile bottom-up synthetic approach to fabricate an emerging class of ND-based hybrid nanostructures in a highly controlled manner, in which the NV centres can be coupled with either plasmonic nanoparticles or excitonic quantum dots. Precise control of critical structural parameters of such hybrid nanostructures, including size, composition, coverage and inter-particle spacing represents a major achievement of our current work, and is a pre-requisite for investigating the underlying physics and further engineering-related optical properties, which is absent in prior attempts^27^,^28^. In particular, the optical properties of both metallic nanoparticles and semiconducting quantum dots are strongly dependent on their size and composition, which offers the opportunity to finely tune the density and energy level of their localized optical states^29^. To that, potential coupling in such hybrid nanostructures, as well as its fine tuning through structural regulation, is further corroborated by the observation of substantial modification of the fluorescence lifetime of NV centres with a strong dependence on structural parameters by both single-particle second-order correlation optical measurements and ultrafast fluorescence lifetime measurements. These as-synthesized hybrid nano structures offer a toolset capable of tailoring properties of NV centres via various coupling interactions and strength, and are fundamentally different from those structures in prior study^9^,^10^,^11^,^12^,^13^,^14^,^15^,^16^,^17^.

## Results

### General synthetic scheme of ND–metal nanoparticles

**Figure 1:**
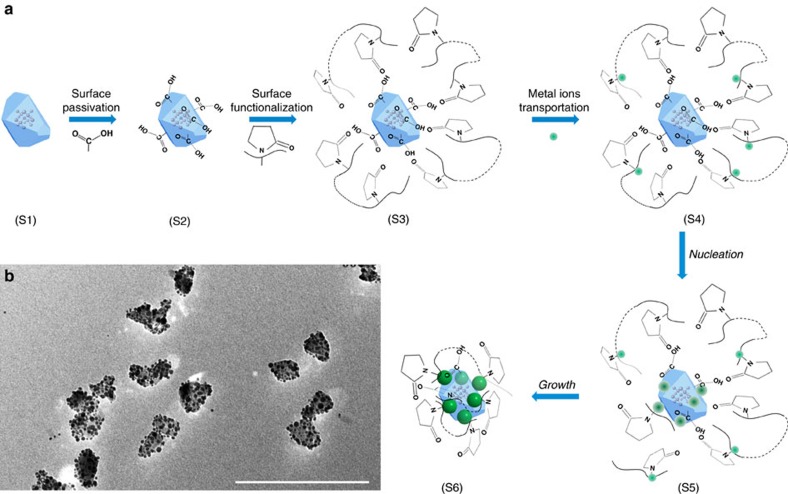
Universal synthetic route for hybrid ND–metal nanoparticles. (**a**) Schematic synthetic paradigm illustrating different growth stages (S1–S6). Stage S1: pure ND. Stage S2: acid treated ND with carboxylic groups. Stage S3: ND with functionalized PVP molecules. Stage S4: anchoring metal ions onto the ND surface. Stage S5: nucleation of metal nanoparticles on the ND surface. Stage S6: growth of metal nanoparticles on the ND surface. (**b**) A typical large-scale TEM image showing excellent dispersion and uniformity of hybrid ND–Ag nanostructures made by following synthetic scheme in **a**. Scale bar, 200 nm.

Figure 1a illustrates the synthetic route for growing metal nanoparticles onto a ND. In brief, we start with size-selected NDs (stage S1) (Supplementary Fig. 1) with extensive acid treatment to passivate the surface of NDs with carboxylic groups that can allow good dispersion in water (stage S2). The carboxylic group terminated ND surface is inert and difficult to directly grow nanoparticles onto. To enable nucleation and control coverage of external coupled functional units, the ND surface is further functionalized with Poly(vinylpyridine) (PVP) molecules (stage S3), of which the pyridyl groups can interact with nonmetallic polar surfaces terminated with carboxyl groups through hydrogen bonding, whereas the nitrogen atoms on the unbound pyridyl groups possesses strong affinity to metal ions (stage S4) desirable for transporting metal ions and guiding nucleation (stage S5) and growth of nanoparticles (stage S6) on the ND's surface^30^. Additionally, the PVP molecules can act as a natural surface ligand for as-grown nanoparticles to ensure excellent dispersion of hybrid nanostructures in solution. Importantly, the surface density of nuclei is simply determined by the density of PVP molecules anchored on the ND's surface and the size of external nanoparticles can be independently controlled by the growth time and temperature, which makes it possible to finely control structural parameters of metal nanoparticles on the surface of the ND.

### Demonstration of fine structural parameter controls

**Figure 2:**
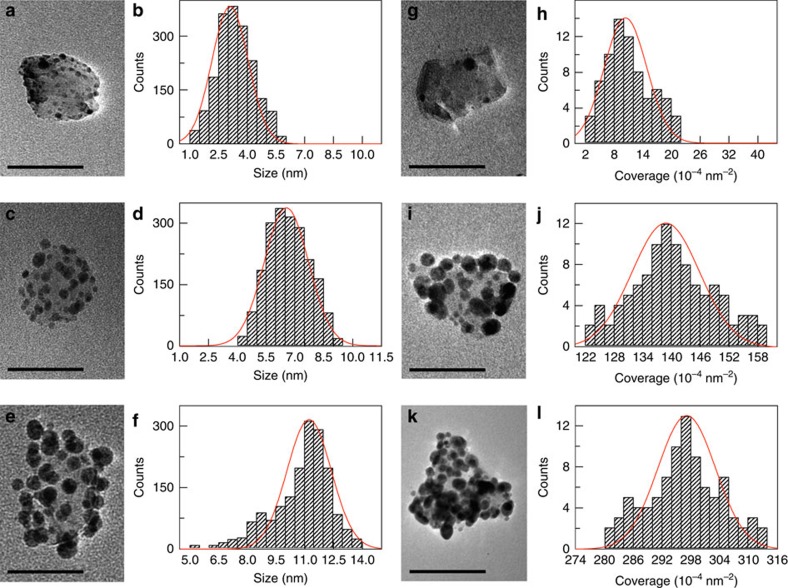
Fine control of size and coverage of Ag nanoparticles in hybrid ND–Ag nanostructures. (**a**–**f**) Size control of Ag nanoparticles with same surface coverage of 0.016±0.002 particles per nm^2^. **a**,**b**, **c**,**d** and **e**,**f** are typical TEM images of a single hybrid nanostructure and their corresponding histogram plot of size distribution, for three different samples to show size evolution. Scale bar of TEM images, 50 nm. Red curve is a Gaussian fit to the histogram plot. (**g**–**l**) Control of surface coverage of Ag nanoparticles possessing same size of 8.6±1.1 nm. **g**,**h**, **i**,**j** and **k**,**l** are typical TEM images of a single hybrid nanostructure and their corresponding histogram analysis of surface coverage distribution, for three different samples to show control of surface coverage of Ag nanoparticles on a ND surface. Scale bar of TEM images, 50 nm. Red curve is a Gaussian fit to the histogram plot.

We have employed ND–Ag hybrid nanostructures as an example to demonstrate the capability of fine control that can be enabled by this synthetic route. The Ag nanoparticle is chosen as an example based on the following three considerations: the Ag nanoparticle possesses a strong localized surface plasmon resonance that can be utilized as a model system to explore coupling to NV centres confined in NDs^31^; Ag has served as an ideal model for understanding metallic nanoparticle growth, exhibiting rich size and shape control in solution^32^; and the Ag ion possesses extremely high Lewis acidity as compared with many other metals and can thus enable different chemical transformation processes for converting Ag to other metals and semiconductors^33^,^34^,^35^,^36^. Figure 1b shows a typical large-scale transmission electron microscope (TEM) image of as-grown ND–Ag hybrid nanostructures to demonstrate uniformity and dispersion of as-synthesized hybrid nanostructures. To evaluate size distribution and coverage of Ag nanoparticles on the surface of NDs we have performed statistical analysis by investigating a large number of nanostructures in the same sample batch. Overall we have achieved sizes ranging from 3 to 30 nm with independent control of surface coverage from a single nanoparticle up to 0.3 particles per nm^2^ on average (Supplementary Fig. 2). Figure 2a–f shows the evolution of size with the same coverage of 0.016±0.002 particles per nm^2^, whereas Fig. 2g–l demonstrate the capability of tuning the coverage for the same size of Ag nanoparticles (8.6±1.1 nm), by both high-resolution TEM imaging and histogram analysis to highlight the synthetic capability that can be enabled by the method illustrated in Fig. 1a. Corresponding large-scale images are presented in Supplementary Fig. 3 to highlight uniformity of as-synthesized hybrid nanostructures, with more in-depth discussion about size and shape distribution in Supplementary Note 1 (see also Supplementary Fig. 4). To unambiguously substantiate our proposed synthetic route in Fig. 1a as well as the assignment of hybrid nanostructures, we have performed one control experiment to confirm that metal Ag nanoparticles are indeed grown onto ND surface rather than physical adsorption by mixing Ag nanoparticles with NDs. Related in-depth discussion on this control experiment is provided in Supplementary Note 2 but, briefly, the mixture samples have shown completely different features from as-synthesized hybrid nanostructures according to our TEM characterizations (Supplementary Fig. 5), which can be safely excluded.

### Coupling NV centres to tunable metal nanoparticles

**Figure 3:**
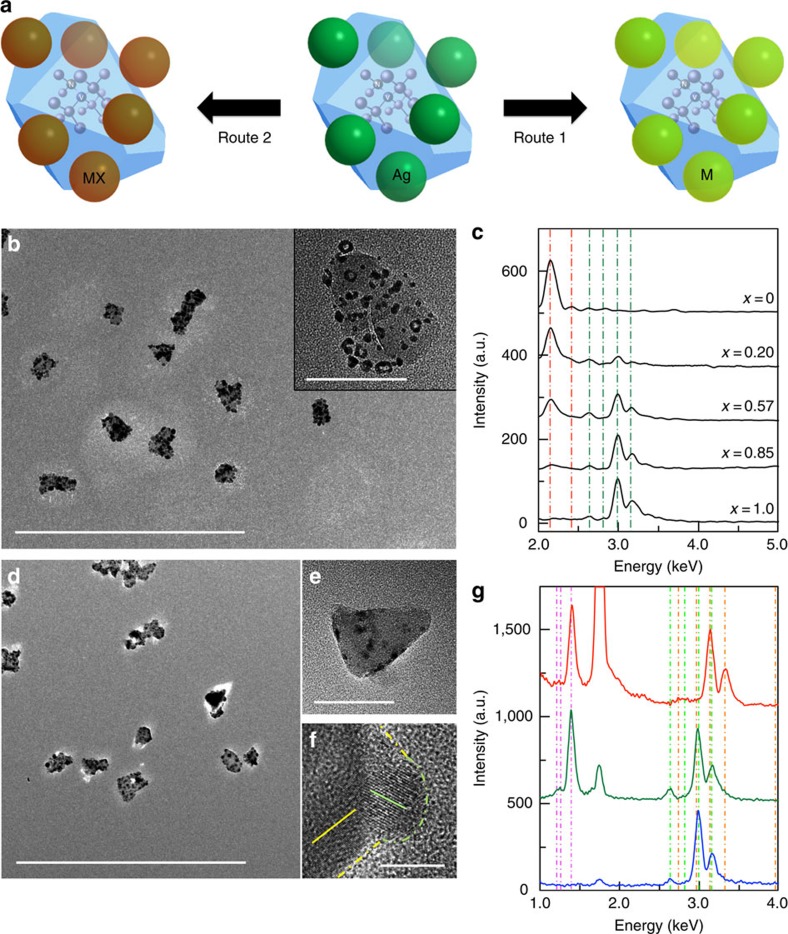
Tuning composition of surface functional units in a hybrid ND based nanostructure. (**a**) Schematic of two chemical transformation processes to convert Ag nanoparticles on the surface of ND to different functional units: metal nanoparticles (M) via Galvanic replacement mechanism (route 1) or semiconductor quantum dots (MX) via ionic exchange mechanism (route 2). X represents a chalcogenide element. (**b**,**c**) Hybrid ND–Au_1−*x*_Ag_*x*_ nanostructures with tunable ratio *x*. (**b**) Typical large-scale TEM image. Scale bar, 500 nm; (inset) TEM image of a single hybrid nanostructure showing the structural characteristics of a Galvanic reaction. Scale bar, 50 nm. (**c**) Evolution of EDS with different *x*, highlighting precise control of composition of metal nanoparticles in a hybrid nanostructure. Vertical green and red dash-dot lines guide the characteristic peaks of Ag and Au elements, respectively. (**d**–**g**) Hybrid ND–CdSe nanostructures possessing monocrystalline CdSe quantum dots. (**d**) Typical large-scale TEM image. Scale bar, 500 nm; (**e**) Typical TEM image of an individual hybrid nanostructure. Scale bar, 50 nm. (**f**) High-resolution TEM image highlighting lattice mismatch at the interface between the ND and CdSe. Solid yellow and green lines highlight lattice orientation of ND and CdSe, respectively. Dashed yellow and green curves highlight boundary of ND and CdSe quantum dot, respectively. Scale bar, 5 nm. (**g**) Evolution of EDS during the growth of ND–CdSe. Blue: ND–Ag; green: ND–Ag_2_Se; red: ND–CdSe. Vertical light green, pink and orange dash-dot lines guide the characteristic peaks of Ag, Se and Cd elements, respectively.

Our fine synthetic control of the size and coverage of Ag nanoparticles on the surface of ND represents a crucial step for tailoring its coupling to the NV centres confined in the ND. Because of its high Lewis acidity, as-grown Ag nanoparticles on a ND's surface can be further converted to various metallic and semiconducting units by different chemical transformation mechanisms (Fig. 3a). Importantly, all materials control as achieved for Ag nanoparticles in a hybrid nanostructure in Figs 1 and 2 (such as size distribution and coverage) can be preserved during a chemical transformation process, which makes it feasible to study various coupling with NV centres in a systematic manner. For example, both localized surface plasmon resonance and excitonic energy depend on size and composition^29^,^37^. Figure 3b,c exemplify growth of ND–Au_1−*x*_Ag_*x*_ nanoparticles with a controlled ratio *x* (*x*=0–1) through a Galvanic replacement reaction (route 1), with corresponding XRD characterization summarized in Supplementary Fig. 6. The large-scale TEM image (Fig. 3b) demonstrates a uniform dispersion of hybrid ND–Au_0.75_Ag_0.25_ nanostructures. A higher resolution image (inset of Fig. 3b) of an individual hybrid nanostructure highlights the appearance of a hollow morphology feature of Au_0.75_Ag_0.25_ nanoparticles that is a signature of the Galvanic replacement mechanism^36^. The ratio *x* can be continuously tuned from 1 (pure Ag) to 0 (pure Au), as shown with the evolution of energy-dispersive X-ray spectroscopy (EDS) measurements presented in Fig. 3c. This tunability can be employed to tailor the localized surface plasmon resonance of metallic nanoparticles on the surface of NDs and to allow the investigation of energy dependent electromagnetic field coupling to the NV centres^37^.

### Coupling NV centres to semiconductor quantum dots

The as-grown external Ag nanoparticles on the surface of ND can also be converted to semiconductor quantum dots with well-defined excitonic features to achieve hybrid ND–semiconductor quantum dot nanostructures by an ionic exchange mechanism (route 2)^33^,^34^, in which the size and distribution of semiconductor quantum dots over the ND's surface are uniquely determined by those of the initial Ag nanoparticles before conversion. Figure 3d–g utilizes ND–CdSe hybrid nanostructures as one example to illustrate the process step by step using high-resolution TEM imaging and elemental analysis, but this synthetic approach should be readily extended to other semiconductors such as CdS, PbSe, ZnS and so on^33^,^34^. A typical large-scale TEM image is presented in Fig. 3d to highlight the excellent dispersion and uniformity of hybrid nanostructures after chemical transformation, with a higher resolution image of an individual hybrid nanostructure shown in Fig. 3e to demonstrate the growth of homogeneous CdSe on the surface of ND. To achieve ND–CdSe, the Ag nanoparticles on the surface of the ND are converted to Ag_2_Se first, followed by cation exchange from Ag to Cd cations. This chemical transformation process as well as assignment of products in each step can also be confirmed by investigating the evolution of XRD characteristic peaks during the reactions (Supplementary Fig. 7). An additional important advantage of our synthetic approach is the ability to achieve monocrystalline semiconducting quantum dots on the crystalline surface of ND without requiring lattice matching. It is worth noting that control of structural defects in materials is important for determining their intrinsic physical properties, which has been an intimidating challenge for traditional epitaxial growth^33^,^34^. By comparing the lattice orientation of the ND and the CdSe quantum dots on its surface as highlighted in the high-resolution TEM images (Fig. 3f; Supplementary Fig. 8), it is evident that monocrystalline CdSe quantum dots can be achieved even though there is a sharp change of lattice orientation at the interface. More enlarged high-resolution TEM images of different ND–CdSe interfaces can be found in Supplementary Fig. 8, which again support the nature of non-epitaxial interface in this type of hybrid nanostructures. According to XRD measurements (Supplementary Fig. 7), we can further assign a monocrystalline wurtzite crystal structure for CdSe quantum dots in our ND–CdSe hybrid nanostructures.

### Growth of spacer to tailor coupling strength

**Figure 4:**
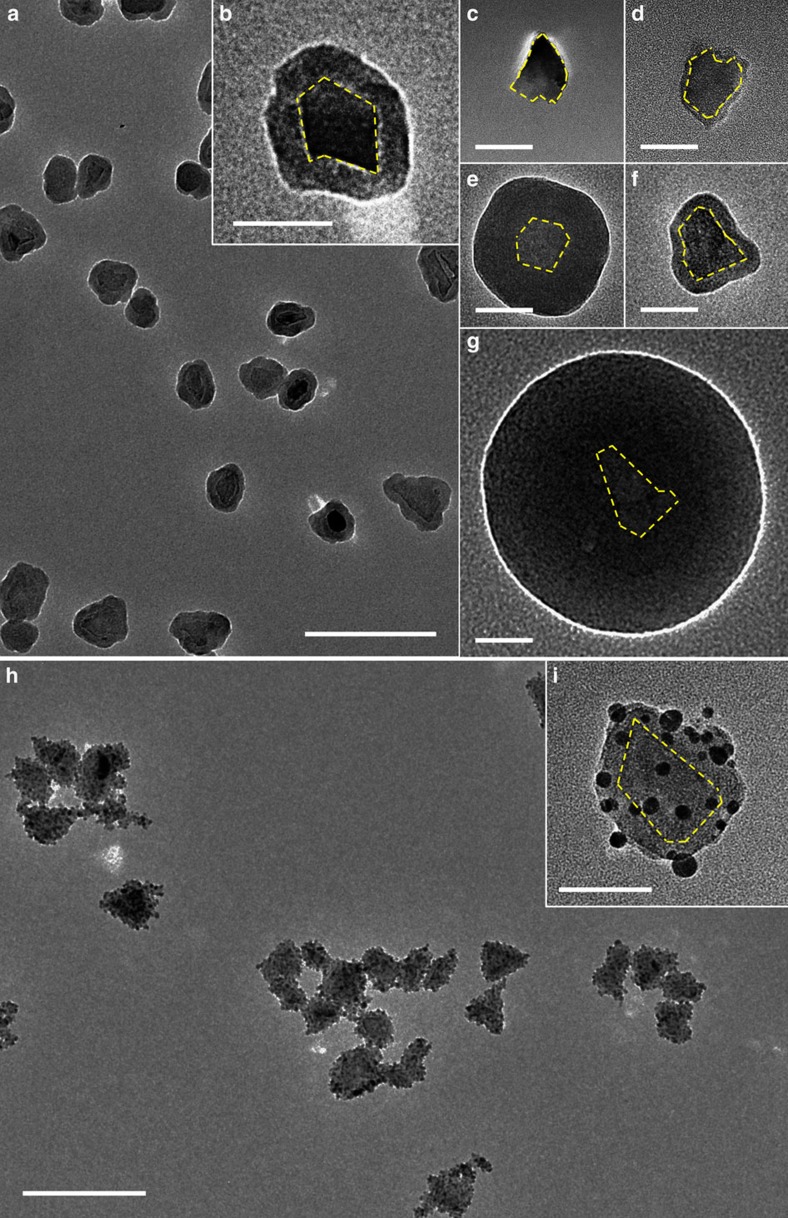
Growth of SiO_2_ spacer with controllable thickness in hybrid ND–SiO_2_–Ag nanostructures. (**a**) Typical large-scale TEM image of ND–SiO_2_ shell. Average thickness of SiO_2_ shell is 14.8 nm. Scale bar, 200 nm. (**b**) Corresponding higher resolution TEM image of an individual ND–SiO_2_ nanostructure. Yellow-dashed curve highlights the interface between the ND and SiO_2_ shell. Scale bar, 50 nm. (**c**–**g**) TEM image of single ND–SiO_2_ shell with different controllable shell thickness of 3.0, 7.5, 12.2, 35.3 and 90.1 nm, respectively. Yellow-dashed curve highlights the interface between the ND and SiO_2_ shell. Scale bar, 50 nm. (**h**) Typical large-scale TEM image of hybrid ND–SiO_2_–Ag nanostructures. Scale bar, 200 nm. (**i**) Corresponding higher resolution TEM image of a single hybrid nanostructure. Yellow-dashed curve highlights the interface between the ND and SiO_2_ shell. Scale bar, 50 nm.

In addition to the size and coverage control of nanoparticles on the surface of a ND, another important structural parameter to tailor fundamental coupling to the NV centres in a ND is the distance between the external units and the NV centres, which can be achieved by adding a wide bandgap spacer between the NV centres and the surface units in a hybrid nanostructure. We have chosen silica (SiO_2_) shells as the spacer to tune the distance between the NV centres and the external surface functional units. The SiO_2_ shell can be grown by controlling hydrolysis of the precursors, such as TEOS, through a Stöber process^38^. The passivated PVP molecules on the surface of ND as illustrated in Fig. 1a (stage S3) can promote the decomposition of TEOS onto ND. One common issue existing in the growth of SiO_2_ shells or nanoparticles is aggregation or poor dispersion in solution. By manoeuvreing the decomposition speed of TEOS, good dispersion of the ND–SiO_2_ shell can be routinely achieved as shown in Fig. 4a (with corresponding typical high-resolution image presented in Fig. 4b), which has been a challenge in prior studies^27^,^39^. Importantly, the thickness of the SiO_2_ shell can be continuously tuned from 3 to 100 nm by simply controlling the growth condition, while still maintaining a good dispersion and uniformity of the shell. A few more examples are highlighted in Fig. 4c–g (see Supplementary Table 1 for different synthetic conditions to achieve different thickness of SiO_2_ shell surrounding the ND core). Only the representative high-resolution TEM images of samples with different SiO_2_ shell thickness are presented here to highlight control of shell thickness because when the SiO_2_ shell increases it becomes difficult to discern core shell features in a large-scale image. However we have provided in Supplementary Fig. 9 a typical large-scale image of sample with thick SiO_2_ shell, to compare with corresponding distribution of thin shell sample in Fig. 4a. A few important conclusions can be obtained. For all of our samples with different SiO_2_ shell thicknesses as shown in Fig. 4b–g, they possess similar uniformity. We have also noticed that when the shell thickness is thin, the shape of the SiO_2_ shell essentially follows that of the ND, while thicker SiO_2_ shells possess a symmetric uniform spherical morphology that is independent of the core ND. This observation might offer a unique synthetic route to achieving a colloidal nanocavity with NV centres in the geometric centre as a new type of nanoscale resonator to enhance photon coupling^40^,^41^.

To demonstrate the feasibility of employing a SiO_2_ shell with tunable thickness as a spacer to control the coupling between the surface functional units and the NV centres, we have shown that Ag nanoparticles can be grown onto a ND–SiO_2_ shell to form complex hybrid ND–SiO_2_ shell-Ag nanostructures. While attempts have been demonstrated to grow metal nanoparticles onto a SiO_2_ surface by utilizing anchoring molecules such as (3-aminopropyl)trimethoxysilane, it has typically led to poor sample dispersion after the growth of nanoparticles due to the cross-linking of anchoring molecules in solution^42^,^43^. To avoid such an aggregation issue, we have first functionalized the SiO_2_ shell with tin chloride (SnCl_2_), in which Sn^2+^ ions can not only be adsorbed onto the negatively charged SiO_2_ surface by electrostatic interaction but also act as a reducing agent of other metal ions such as Ag^+^ (Supplementary Fig. 10)^44^. Figure 4h highlights one example of well-dispersed hybrid ND–SiO_2_ shell-Ag nanostructures with uniform surface coverage of Ag nanoparticles. A typical higher resolution image of an individual hybrid nanostructure (Fig. 4i) can clearly show the ND, silica shell and outermost Ag nanoparticles.

### Fluorescence modification in ND hybrid nanostructures

**Figure 5:**
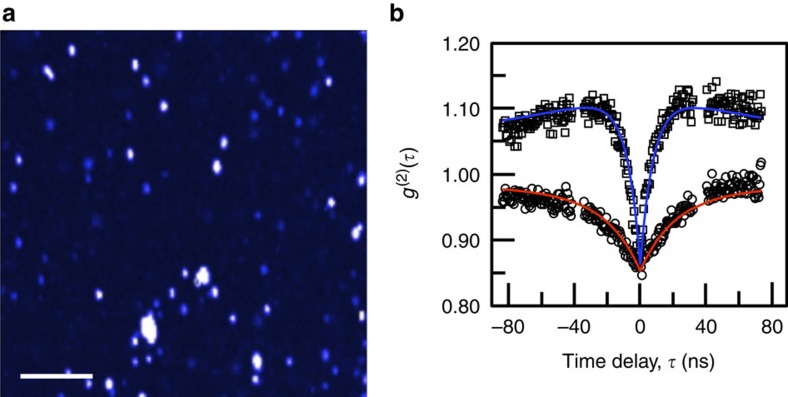
Plasmon-NV coupling in a hybrid ND–Ag nanostructure with 5.0 nm Ag nanoparticles. (**a**), A two-dimensional fluorescence image of hybrid ND–Ag nanostructures. Scale bar, 10 μm. (**b**) Typical autocorrelation (*g*^(2)^(*τ*)) plots of pure ND (circle) and hybrid ND–Ag nanostructure (square). Solid red and blue curves are bi-exponential decay fit to data of ND and ND–Ag, respectively (see Methods). Both data were acquired from the NDs containing same amount of NV centres (six).

**Figure 6:**
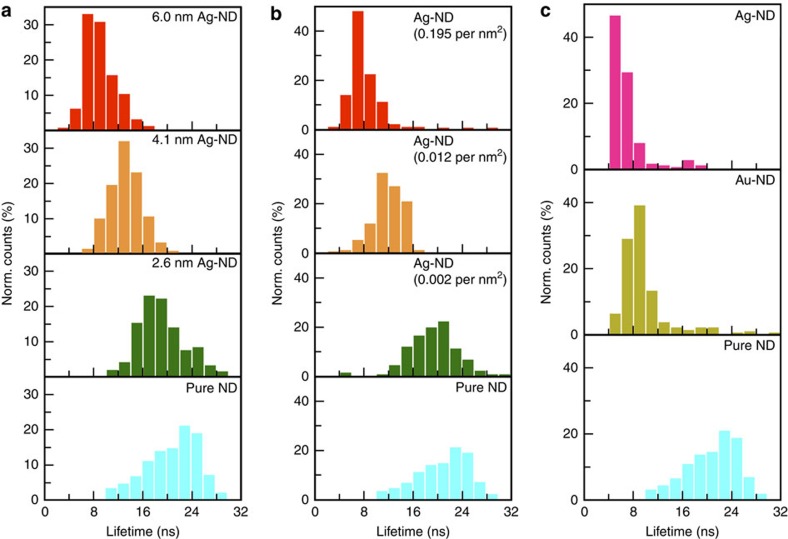
Tailoring plasmon-NV coupling in various hybrid ND–metal nanostructures. (**a**) Dependence of the fluorescence lifetime of NV centres on the size of Ag nanoparticles in a hybrid ND–Ag nanostructure (corresponding materials control presented in Fig. 2a–f). Cyan: pure ND; green: ND–Ag (2.6 nm); orange: ND–Ag (4.1 nm); red: ND–Ag (6.0 nm). All ND–Ag hybrid nanostructures possess same surface coverage of Ag subunits (0.004 particles per nm^2^). (**b**) Dependence of the fluorescence lifetime of NV centres on the coverage of Ag nanoparticles in a hybrid ND–Ag nanostructure (corresponding materials control presented in Fig. 2g–l). Cyan: pure ND; green: 0.002 particles per nm^2^; orange: 0.012 particles per nm^2^; red: 0.195 particles per nm^2^; mean size of Ag subunits in all hybrid ND–Ag nanostructures is 4.5 nm. (**c**) Dependence of fluorescence lifetime of NV centres on the composition of metal nanoparticles in a hybrid ND based nanostructure (corresponding materials control presented in Fig. 3c). Cyan: pure ND; dark yellow: ND–Au; pink: ND–Ag. Surface coverage density of both ND–Au and ND–Ag hybrid nanostructures is 0.008 particles per nm^2^.

Our ability to synthesize ND-based hybrid nanostructures with fine control of structural parameters as demonstrated in Figs 1, 2, 3, 4, represent an important step forward to explore various plasmonic and excitonic coupling with NV centres and to control their optical properties, such as fluorescence lifetime. While the reduction of the fluorescence lifetime for those NV centres coupled to surface plasmon modes of metallic nanostructures has been demonstrated in lithography- or nanomanipulation-fabricated devices^9^,^10^,^11^,^12^,^13^,^14^,^15^,^16^,^17^, our colloidal freestanding hybrid structures are scalable, with more sophisticated and diverse materials control, and allow us to create much smaller but complex structures with tailored optical characteristics than what have been demonstrated by other techniques. A series of single-particle optical measurements from as-synthesized ND–metal hybrid nanostructures are summarized in Figs 5 and 6 to highlight synergistic coupling between the NV centres and metal nanoparticles and its unique tunability through structural regulation. All samples in the current optical study were prepared by spin coating hybrid nanostructures on a glass coverslip which was then covered with a layer of Poly(methyl methacrylate) (PMMA) inside a nitrogen-filled glove box to prevent oxidation of the metal nanoparticles, as metal oxide can form fluorescent nanoclusters on their surface under photoexcitation (see Supplementary Figs 11 and 12 with detailed discussion of suppression of fluorescence of metal oxide in Supplementary Note 3)^45^. Figure 5a shows a typical two-dimensional fluorescence image of hybrid nanostructures consisting of one ND with 5.0 nm sized Ag nanoparticles on the surface, highlighting the as-prepared sample distribution and quality on the substrate to allow identification of individual nanostructures. The intra-particle coupling between the NV centres confined in a ND and metal nanoparticles on the surface is first confirmed by performing autocorrelation (*g*^(2)^(*τ*)) measurements in a Hanbury–Brown–Twiss (HBT) setup (Supplementary Fig. 11). Photon antibunching in the *g*^(2)^(*τ*) measurement not only reveals the non-classical behaviour of the emitter, but also can be used to determine the fluorescence lifetime and number of emitters (*N*) of a fluorescence source^46^. A typical autocorrelation measurement of an individual ND–Ag nanostructure from the sample in the fluorescence map is presented in Fig. 5b. By comparing with data from pure ND with the same number of NV emitters (six for this data set), the coupling between the NV and Ag nanoparticle is evident with the observation of a clear faster decay rate in the ND–Ag hybrid nanostructure with a steeper slope near the dip, suggesting a substantial reduction of the fluorescence lifetime of NV centres when coupled to 5.0 nm Ag nanoparticles.

To demonstrate the unique tunability of emission characteristics of NV centres in a hybrid nanostructure that can be enabled by our as-achieved structural controls, we have performed a more thorough investigation of the fluorescence lifetime of NV centres in a hybrid nanostructure with a femtosecond pulsed Ti:S laser pumped supercontinuum white light as excitation laser source (Supplementary Fig. 13). We have investigated its dependence on size (Fig. 6a), coverage (Fig. 6b) and composition (Fig. 6c) of metal subunits in a hybrid nanostructure. Each histogram plot of the lifetimes in the figures was obtained by measuring more than 100 hybrid nanostructures. For comparison purposes, the fluorescence lifetime measurement of bare ND is also presented. The averaged lifetime of bare ND is 21.3 ns, which is longer than previous value from bulk diamond^46^. However, this is due to the difference in refractive index (*n*) from an NV centre located inside a diamond (*n*=2.4) and our NDs which are embedded in a layer of PMMA layer on top of a glass substrate (both have *n*∼1.5)^47^,^48^. A clear tendency of the variation of fluorescence lifetimes of NV centres can be observed in all three controls. Figure 6a shows the evolution of decay lifetime from the bare NDs to ND–Ag structures when the averaged size of Ag subunits is only slightly increased from 2.6 to 6.0 nm while maintaining constant coverage density (∼0.004 particles per nm^2^). As the Ag nanoparticle size increases, the mean fluorescence lifetime decreases to 9.0 ns, a 2.4-fold decrease in the fluorescence lifetime as compared with bare NDs. This evolution can be attributed to the increased intensity of the surface plasmon resonance of metal nanoparticles with increasing size^22^. Additionally, when increasing the coverage density by about two orders while keeping the same size Ag nanoparticles (4.5 nm), the fluorescence lifetime of NV centres is reduced consistently and an ∼3.5-fold reduction in lifetime can be identified. Qualitatively, this behaviour can be understood, as increasing the number of plasmonic metal nanoparticles on the ND surface increases the likelihood of an NV centre in the ND coupling to the surface plasmon mode of the Ag nanoparticles. Finally, we have observed that the composition of metal nanoparticles coupling to the NV centres also plays a key role in tailoring emission properties of NV centres. We have particularly compared the coupling between the NV centres and pure Ag and pure Au nanoparticles in Fig. 6c, respectively. As demonstrated in Fig. 3b,c, since the Au subunits are converted *in situ* from the Ag subunits in a hybrid nanostructure, this process can ensure no variation of volume and coverage density of metal nanoparticles when we compare the ND–Ag with ND–Au samples. As a result, the difference shown in Fig. 6c is only due to the change of composition. Au and Ag nanoparticles have been demonstrated to possess distinct surface plasmonic bands^22^, thus the corresponding energy overlap between the NV centres and the plasmonic nanoparticles results in the observed difference of fluorescence lifetimes. While a thorough understanding of the underlying physics behind finely tailored coupling between metal nanoparticles and NV centres requires more controlled experiments as well as sophisticated modelling, the clear tendency revealed in Fig. 6a–c suggests that stronger resonant coupling with NV centres confined in ND and a dramatic improvement of quantum emission should be expected^37^, by fine tuning the structural parameters of ND based hybrid nanostructures as achieved in Figs 2, 3, 4. Employing the synthetic ability achieved in our current study, it is possible to explore the coupling between the NV centre and other nanostructured systems that may lead to improved nanoscale sensors. In the future, hybrid nanostructures consisting of smaller NDs and larger metallic nanoparticles may allow us to investigate single emitters non-linearly coupled to surface plasmon modes^49^.

## Discussion

We have developed a bottom-up synthetic strategy to create hybrid nanostructures that can couple quantum emitters in ND with external functional nanoscale units. Our synthetic strategy allows facile control of important structural parameters that are crucial for tailoring fundamental coupling properties, including size, surface coverage density, composition and spacing. Even though we use commercial NDs with an average size of 40 nm to demonstrate synthetic control of ND-based hybrid nanostructures, our method does not depend on any specific type of NDs to synthesize all of the hybrid nanostructures achieved in this work. In the future, using smaller NDs or those containing single NV centre will open new avenues for the study of physics in these hybrid nanostructures. For example, NV centres confined in a smaller volume can have their location more precisely determined, which should be critical in the future for a thorough understanding of the nature of the coupling effect presented in Figs 5 and 6 and for further evaluating radiative and non-radiative enhancement (Supplementary Note 4).

Compared with all existing methods to create coupling between NV centres and plasmonic/photonic structures, a few advantages of our approach can be immediately identified. First, our hybrid nanostructures possess exceptional structural tunability that is crucial for modifying the interaction with NV centres. Second, a large quantity of hybrid nanostructures with exceptional quality can be achieved in one batch of solution synthesis, in contrast to fabrication at the level of individual nanostructures. Third, our hybrid nanostructure is freestanding and can be easily combined with various bottom-up assembly strategies for functional device scale-up. In particular, NV centres have been recently demonstrated to have promising applications in micro fluidics and biological living cell systems^25^,^50^,^51^. Our synthesized ND-based hybrid nanostructures can serve as a structural scaffold for self-assembling bottom-up hybrid quantum devices. All these unique features of our as-synthesized hybrid nanostructures offer a critical step toward the ultimate control of related optical properties of nanoscale NV emitters. Indeed, by tuning related structural parameters we have successfully demonstrated that the emission characteristics of the NV centres can be tailored by controlling the size, coverage and composition of coupled metal subunits, with an observed enhancement of the decay rate.

Our achievement of ND-semiconducting quantum dot hybrid nanostructures should allow the unique opportunity to study tunable energy transfer between the NV centre and quantum-confined excitons through size control of the quantum dots, which can be challenging for other systems like dye molecules^52^. To explore this opportunity in our as-synthesized hybrid nanostructures, we have evaluated the FRET distance in ND–CdSe nanostructures, which is defined as the distance at which the FRET efficiency from the NV to CdSe is 50% as a function of the CdSe excitonic energy^52^. We have found that FRET can occur in ND–CdSe hybrid nanostructures (Supplementary Fig. 14), showing clear dependence of the excitonic energy of semiconductor quantum dots that can be uniquely tuned by tailoring the size of the quantum dots. This calculation also provides materials design guidelines for our hybrid ND–semiconductor nanostructures to experimentally observe FRET in the future. In addition, ultrafast spin excitation of colloidal quantum dots has been successfully demonstrated using an optical orientation technique, which might result in dipolar spin interactions between the NV centre and semiconductor quantum dots in our hybrid nanostructures^22^,^53^. Our work opens a rich toolbox to engineer properties of quantum emitters from the bottom-up and offers a high-level control of the structure formation while overcoming the limitations of previous attempts^19^,^54^.

## Methods

### Characterizations of ND-based hybrid nanostructures

All samples for TEM characterization are prepared by adding one drop of the ND-based hybrid nanostructures in ethanol onto 300 mesh copper grids with carbon support film (Ted Pella, 01820). JEOL 2100F and JEM 2100 LaB6 TEM are applied for size, morphology and ensemble EDS (Oxford's INCA 100) characterizations. Analysis of size distribution is typically acquired by counting >50 hybrid nanostructures. Analysis of coverage density is typically acquired by counting >70 hybrid nanostructures. Uncertainty/error bar of the size distribution and coverage of nanoparticles is defined as the standard deviation in a statistical analysis of TEM images (detailed data analysis is available in Supplementary Notes 1 and 2).

### Surface functionalization of NDs with PVP molecules

In a typical functionalization process of ND's surface, 0.040 mg of NDs are mixed with 4.0 ml of PVP aqueous solution with concentration of 0.020 g ml^−1^. After being stirred for 12 h, NDs are washed out with deionized water by centrifugation at 12,000 r.p.m. for 10 min for three times, and are re-dispersed in 0.10 ml of deionized water.

### Typical synthesis of ND–Ag hybrid nanostructures

Growth of Ag nanoparticles onto ND is performed in ethanol solution with PVP molecules as a reducing agent. As-prepared PVP functionalized NDs are added into 4.0 ml of ethanol solution containing PVP with desirable concentration, followed by addition of AgNO_3_ solution drop by drop with vigorous hand shaking. The solution mixture is kept at a desirable temperature in the dark to allow full reduction of AgNO_3_ as well as growth of Ag nanoparticles. The size and coverage of Ag nanoparticles can be controlled by adjusting the concentration of PVP molecules, the amount of AgNO_3_, growth time and temperature. After the growth of Ag nanoparticles, the ND–Ag is washed out and cleaned with ethanol by centrifugation at 12,000 r.p.m. for 10 min for three times for further characterization and reaction.

### Typical synthesis of ND–Au_1−*x*_Ag_*x*_ hybrid nanostructures

The ND–Au_1−*x*_Ag_*x*_ is obtained through Galvanic replacement of ND–Ag with HAuCl_4_. The ratio *x* can be tuned by adjusting the ratio between ND–Ag and HAuCl_4_. In a typical synthesis, as-synthesized ND–Ag is dispersed into 5 ml of PVP aqueous solution (2.0 mg ml^−1^), followed by addition of HAuCl_4_ solution with desirable concentration. The solution was kept at room temperature for 6 h, followed by a thorough cleaning by centrifugation three times.

### Typical synthesis of ND–CdSe hybrid nanostructures

To synthesize ND–CdSe hybrid nanostructures, the ND–Ag is converted to ND–Ag_2_Se first by using freshly prepared NaHSe solution as Se precursor (a detailed preparation procedure of NaHSe precursor is available in the Supplementary Methods). In this chemical transformation, the ND–Ag is dispersed into 4.0 ml of ethanol and is degassed with dry nitrogen gas at 60 °C for 15 min, followed by the addition of 0.20 ml of the NaHSe solution. After that, the reaction is stopped and the sample is washed out and cleaned with ethanol by centrifugation at 12,000 r.p.m. for 10 min three times. The ND–Ag_2_Se sample is re-dispersed in 0.2 ml ethanol for characterization and further reaction of ND–CdSe. The ND–CdSe can then be obtained by cation exchange of ND–Ag_2_Se nanostructures. For example, the ND–Ag_2_Se nanostructures are dispersed in 4 ml of ethanol and is degassed with dry nitrogen gas for 30 min, followed by the addition of 0.25 ml of Cd(NO_3_)_2_ (25 mg ml^−1^) and 50 μl of TBP molecules. The solution is kept at room temperature for 30 min. The ND–CdSe is then washed out and cleaned by centrifugation at 12,000 r.p.m. for 10 min three times and is re-dispersed in ethanol for characterization.

### Typical synthesis of ND–SiO_2_ shell

The synthesis starts with NDs functionalized with PVP molecules (stage S3 in Fig. 1a). As-prepared ND/PVP is re-dispersed in 10 ml of ethanol with 4% ammonia solution. A desired volume of TEOS is added to the solution under vigorous stirring. The mixture is then kept at room temperature for 12 h for complete hydrolysis of TEOS and growth of the SiO_2_ shell. The obtained ND–SiO_2_ shell is washed out and cleaned with ethanol by centrifugation at 12,000 r.p.m. for 10 min three times and is re-dispersed in ethanol for further characterization and reaction. Synthetic conditions for samples in Fig. 4a–g are summarized in Supplementary Table 1.

### Typical synthesis of ND–SiO_2_–Ag hybrid nanostructures

To grow Ag nanoparticles onto ND–SiO_2_ shell nanostructure, the surface of ND–SiO_2_ shell is first functionalized with Sn^2+^ ions by mixing with 3 ml of SnCl_2_ solution (0.04 mM) under vigorous stirring for 1 h. The ND–SiO_2_ shell is then washed with deionized water by centrifugation at 12,000 r.p.m. for 10 min three times and is re-dispersed in 0.20 ml of deionized water. The 1.50 ml of freshly prepared Tollens' reagent (10 mM) is mixed with the Sn^2+^ functionalized ND–SiO_2_ shell, and the reaction continues for 30 min under sonification to grow Ag nanoparticles. The ND–SiO_2_–Ag is washed and collected by centrifugation at 12,000 r.p.m.s for 10 min and is then re-dispersed in water.

### Fluorescence imaging, autocorrelation and fitting

In our lab-built confocal microscope (Supplementary Fig. 11), a 532 nm continuous wave solid-state laser is used to excite single NDs while its fluorescence is collected by a 100 × oil immersion objective lens (Olympus numerical aperture (NA) 1.49). Excitation light is removed by a 600 nm long-pass filter in the detection path and the remaining fluorescence is focused into a 50:50 fibre optic beamsplitter attached to two avalanche photodiodes (APD). A fast steering mirror is used to raster scan the excitation beam and create a two-dimensional fluorescence map of the sample and to identify individual nanoparticles. To determine the number of NV centres confined in NDs, we have performed autocorrelation (*g*^(2)^(*τ*)) measurement of individual nanoparticles in a HBT setup. During this measurement, the fluorescence is monitored and the position of the nanostructure is tracked using a software feedback loop to ensure stability of measurement over an extensive period. The measured autocorrelation spectrum can be fit to a bi-exponential decay: 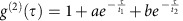, where *a* and *b* are coefficient, and the lifetimes *t*_1_ and *t*_2_ represent the lifetime of the fluorescence of the excited state and of the metastable shelving state of the NV centre, respectively. The number of emitters *N* can be obtained from the value 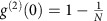.

### Fluorescence lifetime measurements, fitting and analysis

In our lab-built confocal microscope (Supplementary Fig. 11), the laser source in our fluorescence lifetime measurements is provided by using a 10 nm bandwidth filter centred ∼532 nm to select light from femtosecond supercontinuum white light, which is generated by focusing a femtosecond pulsed Ti:S laser (Spectra Physics, 80 MHz repetition rate) into a photonic crystal fibre. Fluorescence is collected with an APD, and lifetime curves are collected using a time correlated single photon counting card (Becker & Hickl). Lifetime curves are fit with a bi-exponential curve (Supplementary Fig. 13) with detailed discussion available in Supplementary Note 4.

### Data availability

All relevant data are available from the authors on request.

## Additional information

**How to cite this article:** Gong, J. *et al*. Nanodiamond-based nanostructures for coupling nitrogen-vacancy centres to metal nanoparticles and semiconductor quantum dots. *Nat. Commun.* 7:11820 doi: 10.1038/ncomms11820 (2016).

## Supplementary Material

Supplementary Information Supplementary Figures 1-14, Supplementary Table 1, Supplementary Notes 1-4, Supplementary Methods and Supplementary References
