# Supplementary material for: Nanodiamond-based nanostructures for coupling nitrogen-vacancy centres to metal nanoparticles and semiconductor quantum dots
Source: Nat Commun. 2016 Jun 8;7:11820. doi: 10.1038/ncomms11820 (PMC4899845; doi:10.1038/ncomms11820)
Supplement: Supplementary Information — Supplementary Figures 1-14, Supplementary Table 1, Supplementary Notes 1-4, Supplementary Methods and Supplementary References [file ncomms11820-s1.pdf]

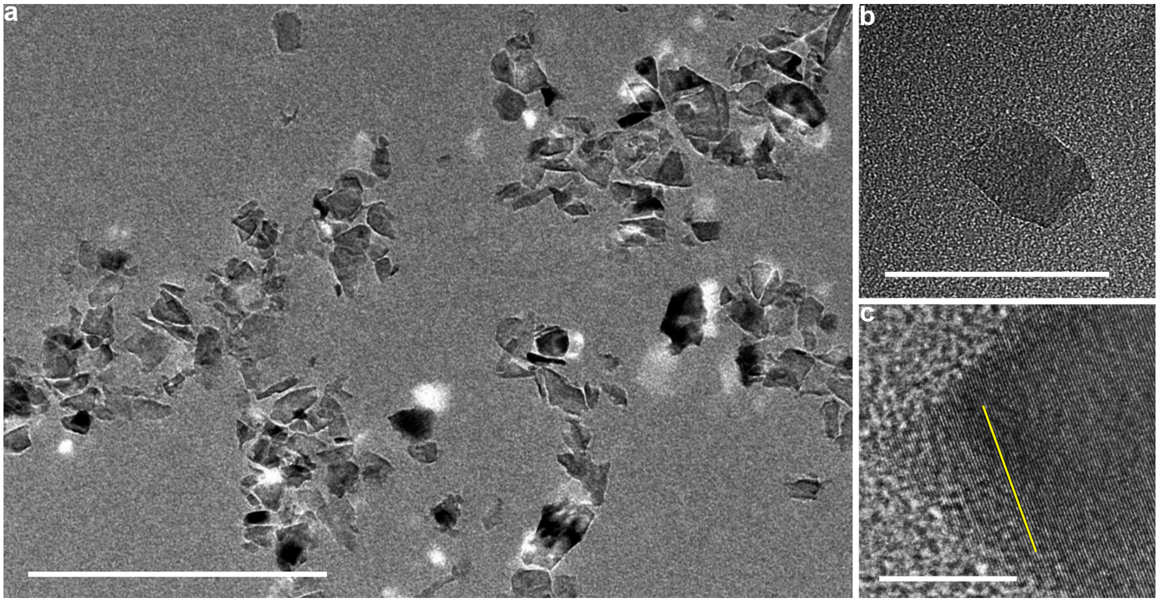

Supplementary Figure 1 | Characterization of pure NDs. a, Typical large-scale TEM image of commercial NDs with an average size of 40 nm. Scale bar, 500 nm. **b**, TEM image of a single ND. Scale bar, 100 nm. As shown in the image, the morphology of NDs is a plate-like shape. **c**, High-resolution TEM image of ND, manifesting crystalline lattice. Yellow line highlights lattice orientation. Scale bar, 2 nm.

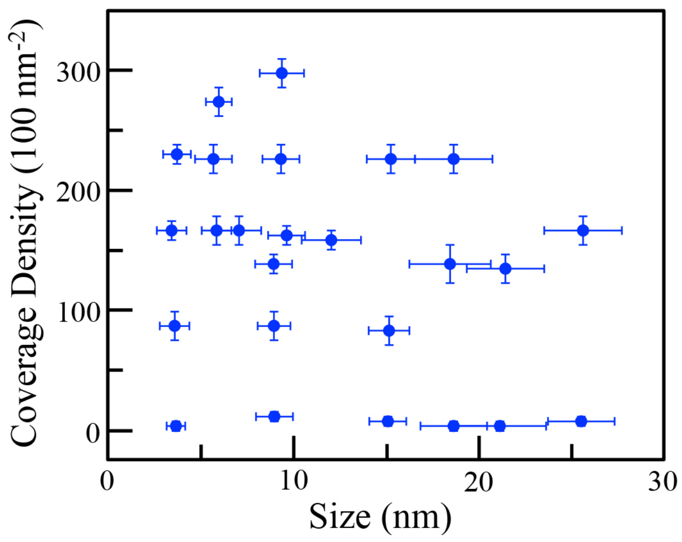

Supplementary Figure 2 | Summary of tunable structural parameters in ND-Ag. All data are acquired from samples prepared by following synthetic paradigm in Figure 1a. Error bars of size and coverage density are determined by the standard deviation in a statistical analysis of related TEM images. Analysis of size distribution is typically acquired by counting more than 50 hybrid nanostructures. Analysis of coverage density is typically acquired by counting more than 70 hybrid nanostructures. See also Supplementary Note 1 for detailed discussion.

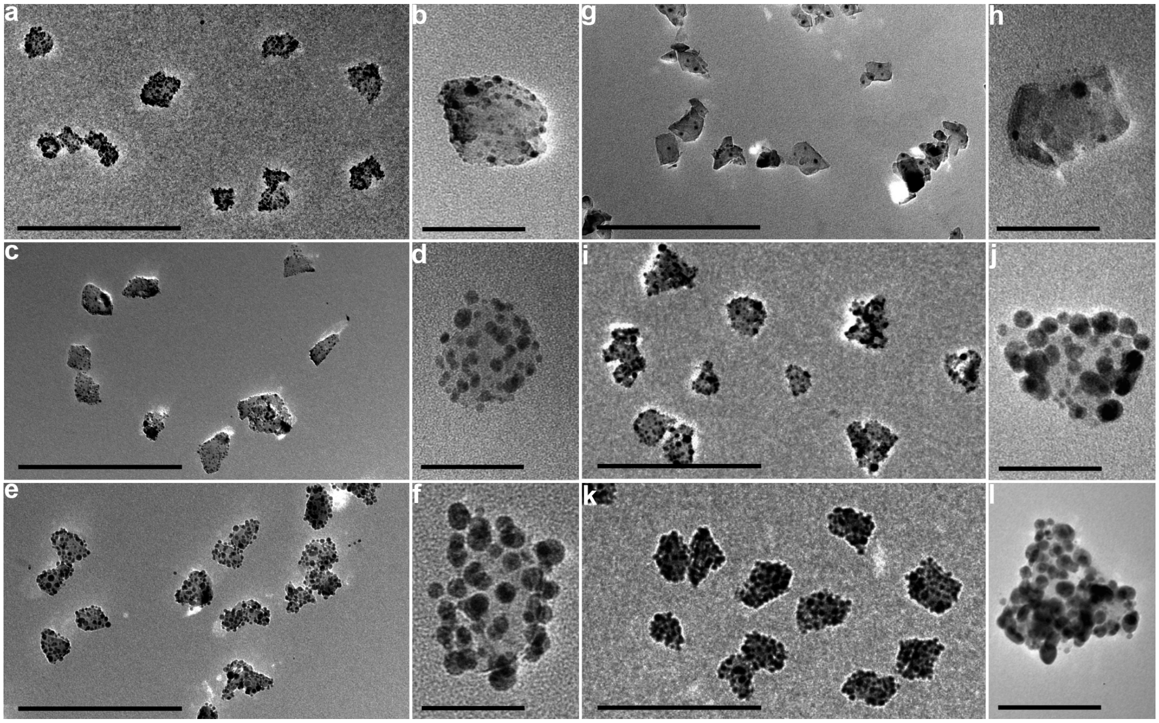

Supplementary Figure 3 | Large-scale TEM characterization for samples presented in Figure 2 in the main text. (**a**, **b**), (**c**, **d**) and (**e**, **f**) correspond to samples in (**a**, **b**), (**c**, **d**) and (**e**, **f**) in Figure 2 in the main text, respectively. (**g**, **h**), (**i**, **j**), and (**k**, **l**) correspond to samples in (**g**, **h**), (**i**, **j**), and (**k**, **l**) in Figure 2 in the main text, respectively. For the purpose of referencing, high-resolution images presented in the main text are also shown in this figure. Scale bar for (**a**, **c**, **e**, **g**, **i**, **k**), 200 nm. Scale bar for (**b**, **d**, **f**, **h**, **j**, **l**), 50 nm.

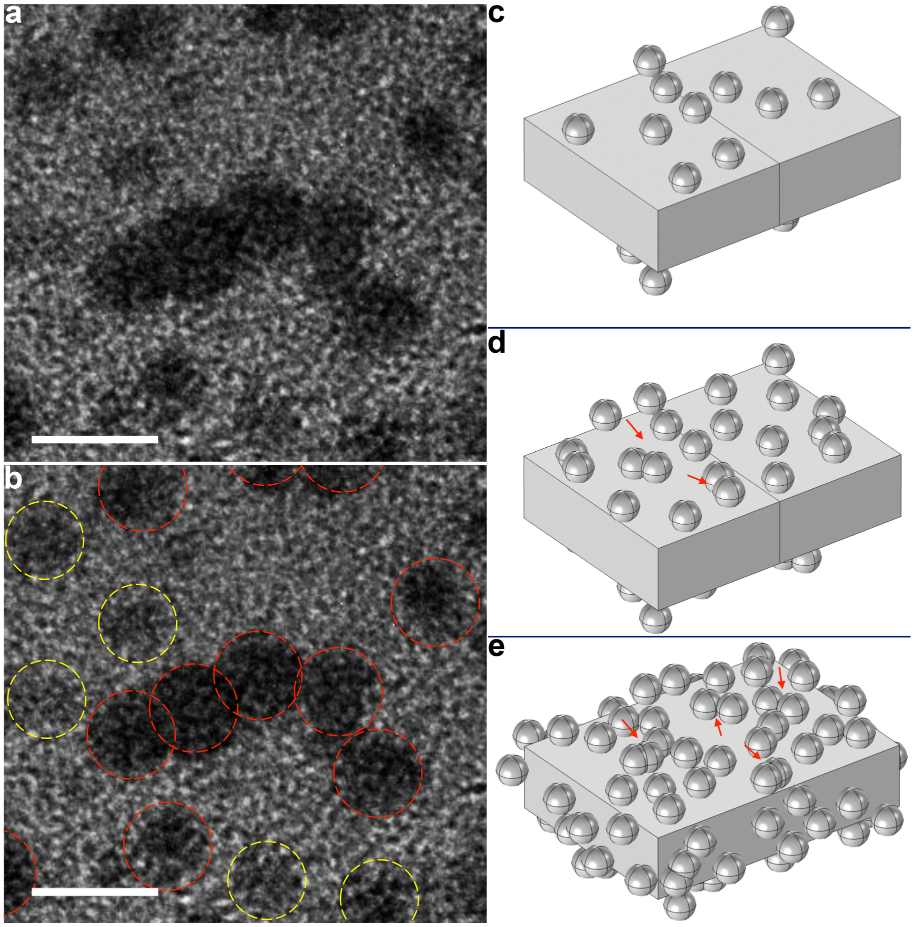

Supplementary Figure 4 | Metal nanoparticles on ND surface. a, TEM image of one selected ND surface area with as-grown Ag nanoparticles. Scale bar, 20 nm. **b**, Same image as **a** but Ag nanoparticles are marked by red (top surface) and yellow (bottom surface) dashed circles. Scale bar, 20 nm. **c-e**, Simulation of Ag nanoparticles on ND surface with low, medium and high surface coverage density, respectively. Red arrows highlight touching of neighboring nanoparticles in the scenario of medium and high coverage. See also Supplementary Note 1 for detailed discussion.

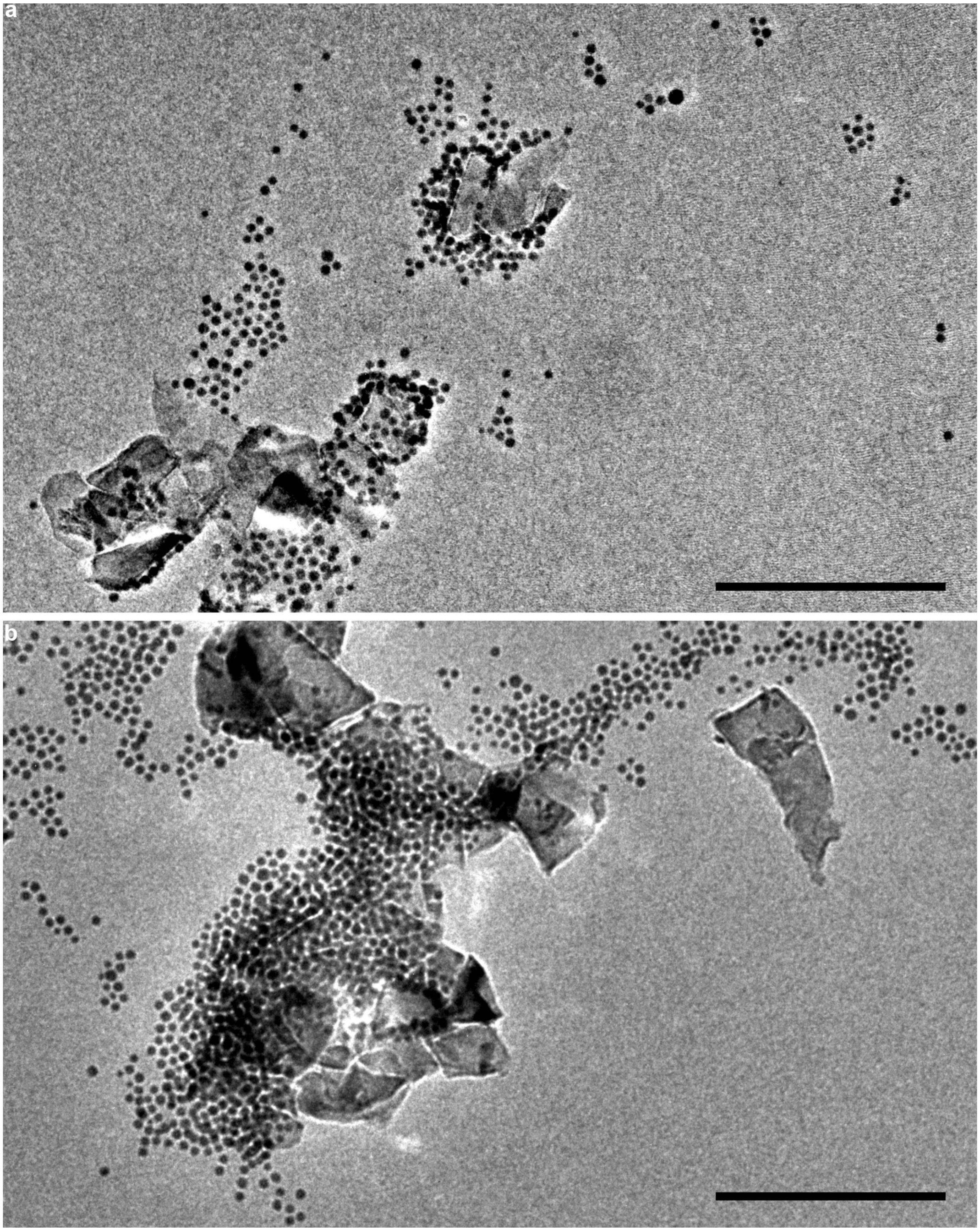

Supplementary Figure 5 | Mixtures of Ag nanoparticles and NDs. a, Typical TEM image of sample with low mixture ratio of Ag nanoparticles to NDs. **b**, Typical TEM image of sample with high mixture ratio of Ag nanoparticles to NDs. Scale bar, 100 nm. See also Supplementary Note 3 for detailed discussion.

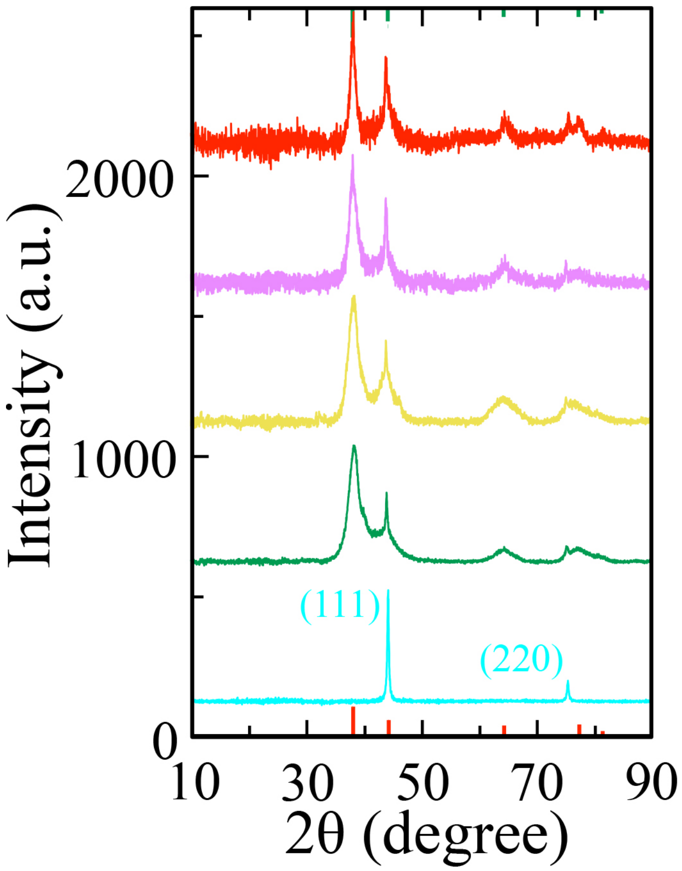

Supplementary Figure 6 | XRD characterization of ND-$Au_{1-x}Ag_x$ hybrid nanostructures. For comparison, XRD of pure ND is presented (Cyan) with two peaks assigned for (111) and (220), respectively. Green, ND-Ag (x=1); Yellow, x=0.85; Purple, x=0.20; Red, ND-Au (x=0). XRD patterns of bulk Au (red vertical lines shown in the bottom axis, JCPDS# 14-0072) and Ag (green vertical lines shown on the top axis, JCPDS# 04-0783) are also provided for reference and comparison. Because Au and Ag have very similar lattice structures, their difference cannot be discerned in the spectra of hybrid nanostructures. However, the presence of XRD characteristic peaks of both ND and Au/Ag confirms our assignment of hybrid nanostructures.

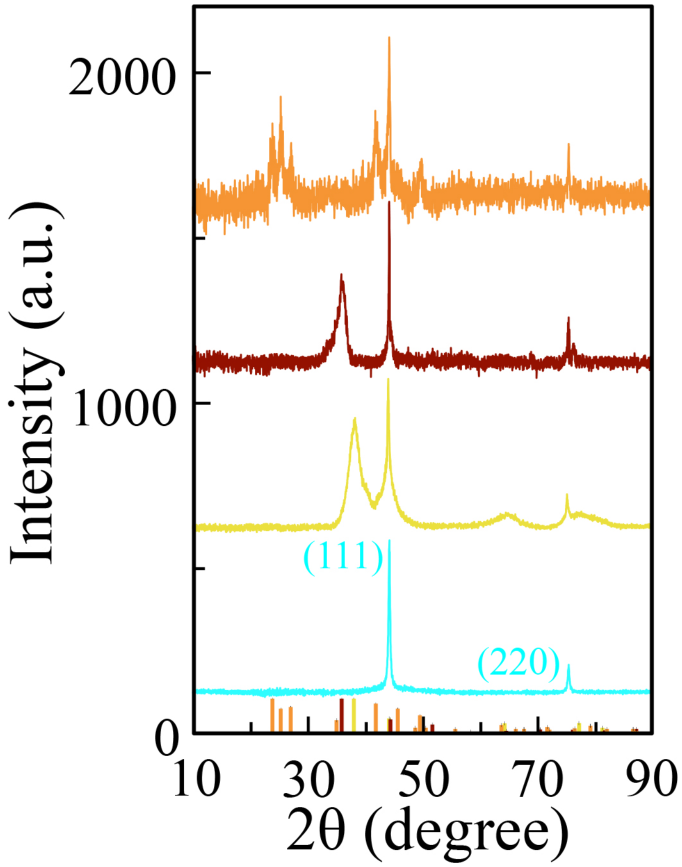

Supplementary Figure 7 | XRD characterization of chemical transformation from ND-Ag to ND-CdSe. For comparison, XRD of pure ND is also presented (Cyan) with two peaks assigned for (111) and (220), respectively. Yellow, ND-Ag; Brown, ND-Ag_2Se; Orange, ND-CdSe. XRD patterns of bulk Ag (yellow vertical lines, JCPDS# 04-0783), cubic Ag_2Se (brown vertical lines, JCPDS# 76-0135), wurtzite CdSe (orange vertical lines, JCPDS# 08-0459) are also provided for reference and comparison. Evolution of XRD characteristic peaks during this chemical transformation process confirms a complete chemical transformation can be achieved in our synthesis with the absence of XRD features from the prior steps. Importantly, both XRD and TEM characterizations clearly support monocrystalline feature of semiconductor quantum dots, and wurtzite crystal structure can be assigned for CdSe quantum dots.

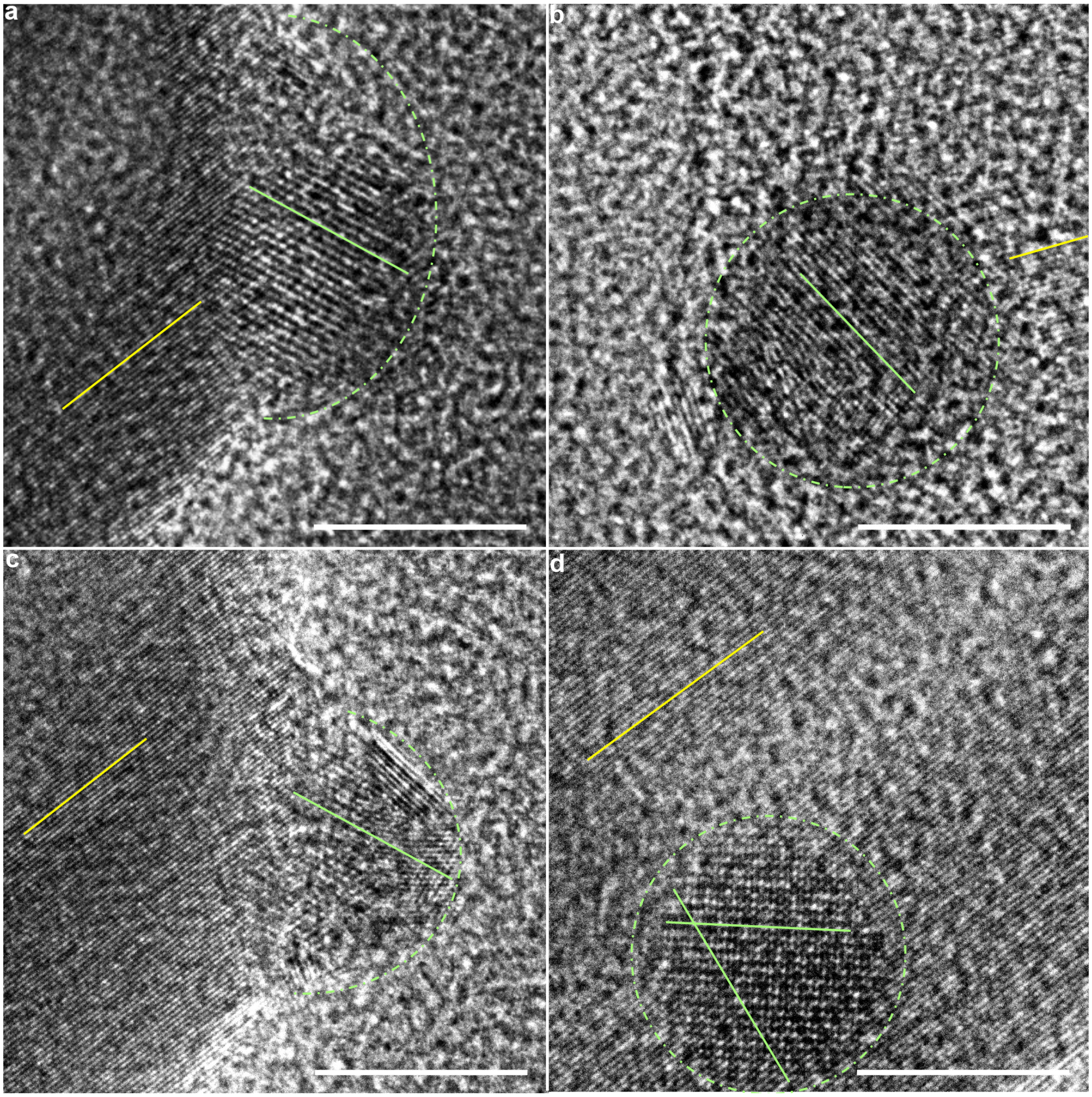

Supplementary Figure 8 | Monocrystalline CdSe quantum dots in ND-CdSe. a-d, High-resolution TEM image collections of ND-CdSe interface to highlight both monocrystalline feature of CdSe quantum dots and non-epitaxial interface between ND and CdSe. Scale bar, 5 nm. Solid yellow and green lines highlight lattice orientation of ND and CdSe, respectively. Green dash-dot curve marks CdSe quantum dots.

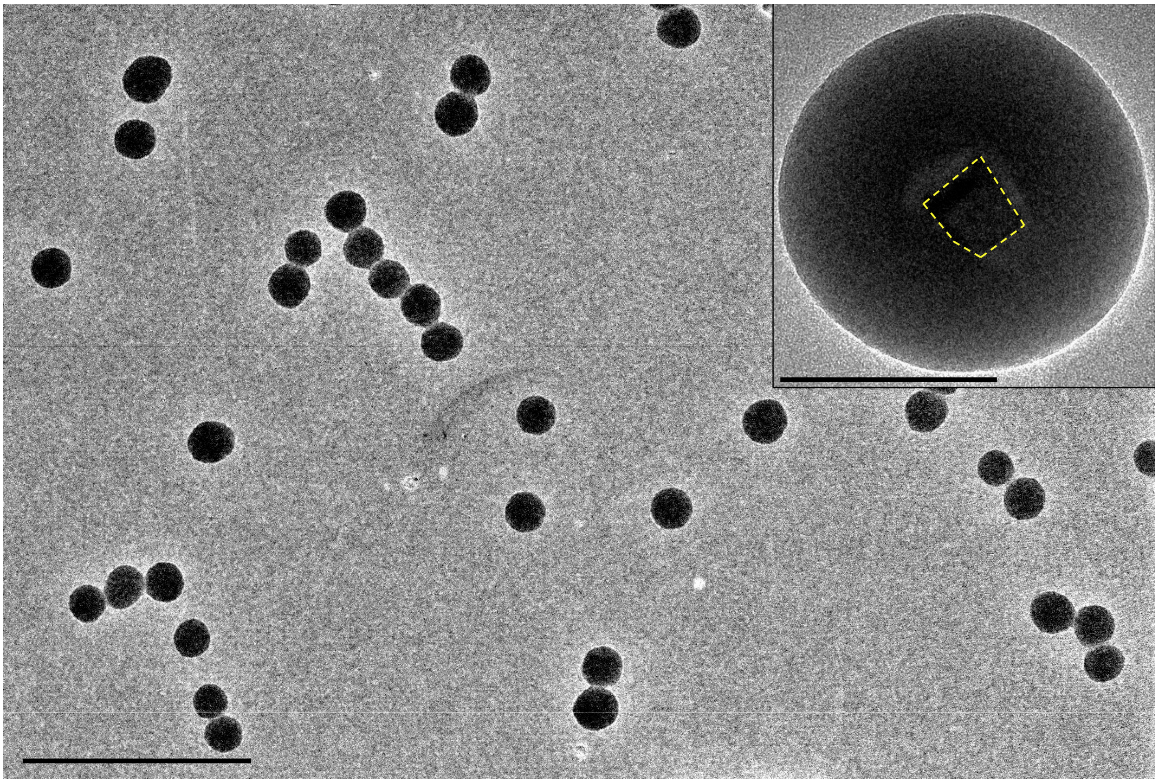

Supplementary Figure 9 | ND-SiO_2 with thick SiO_2 shell. Typical large-scale TEM image. Scale bar, 1 µm. (**Inset**) A higher resolution TEM image of an individual nanostructure with core ND marked by yellow dashed lines. Scale bar, 100 nm.

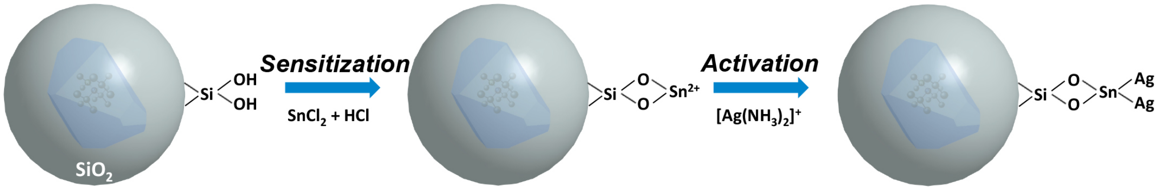

Supplementary Figure 10 | Schematic synthetic paradigm of ND-SiO_2-Ag. The growth starts with ND-SiO_2, followed by two main synthetic steps: sensitization of silica shell surface, and immobilization of Ag ions onto the silica surface.

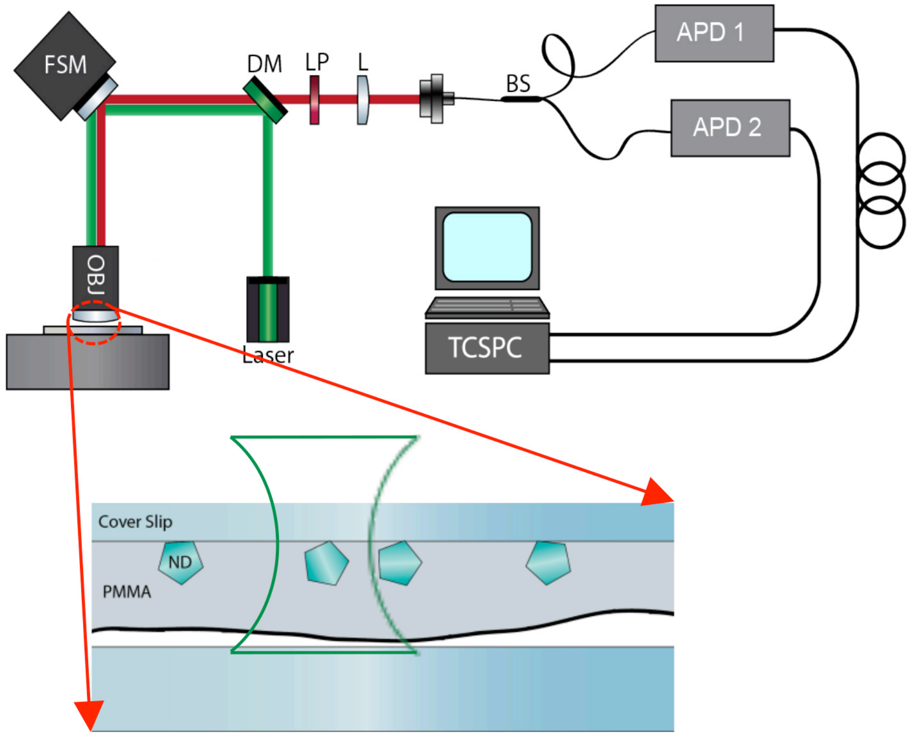

Supplementary Figure 11 | Schematic of single particle optical measurement. Both autocorrelation $g^{(2)}(\tau)$ measurement in a HBT configuration and ultrafast fluorescence lifetime measurement can be achieved for single hybrid ND based nanostructure.

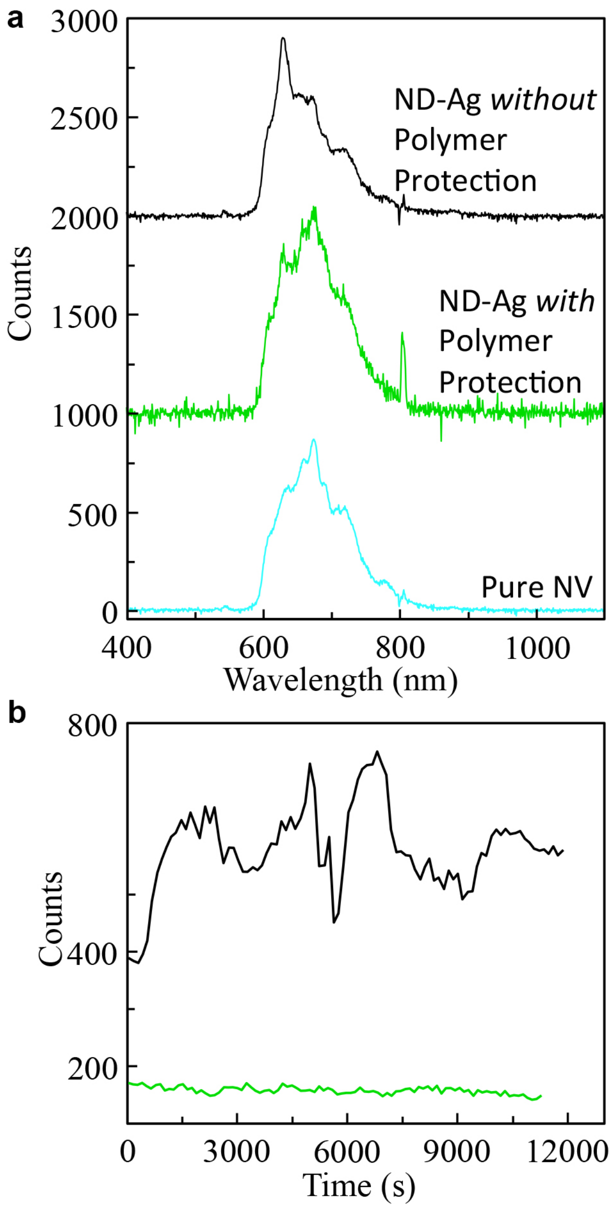

Supplementary Figure 12 | Suppression of fluorescence of Ag oxide in ND-Ag. a, Comparison of fluorescence spectra among different samples. Spectra of ND-Ag hybrid nanostructures are vertically shifted for clarity purpose. **b**, Comparison of time trace of fluorescence intensity in ND-Ag without (black) and with protection (green).

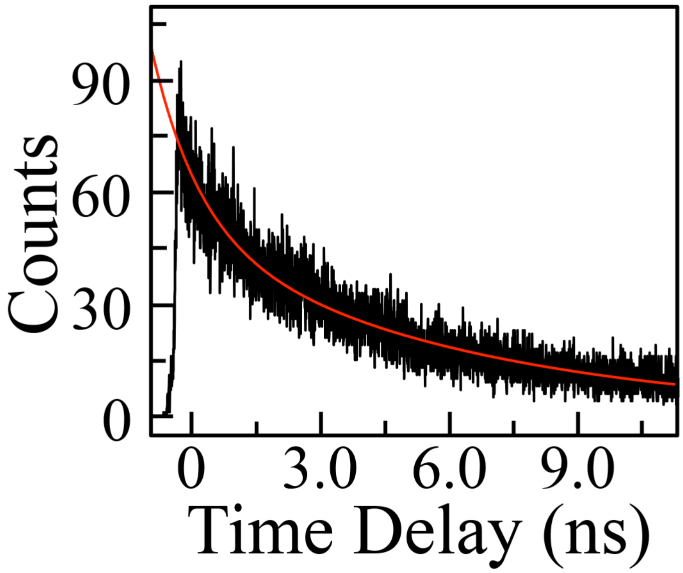

Supplementary Figure 13 | Typical fluorescence lifetime trace and fitting. Black curve: experimental data. Red curve: fit (see also Supplementary Note 4 for detailed discussion).

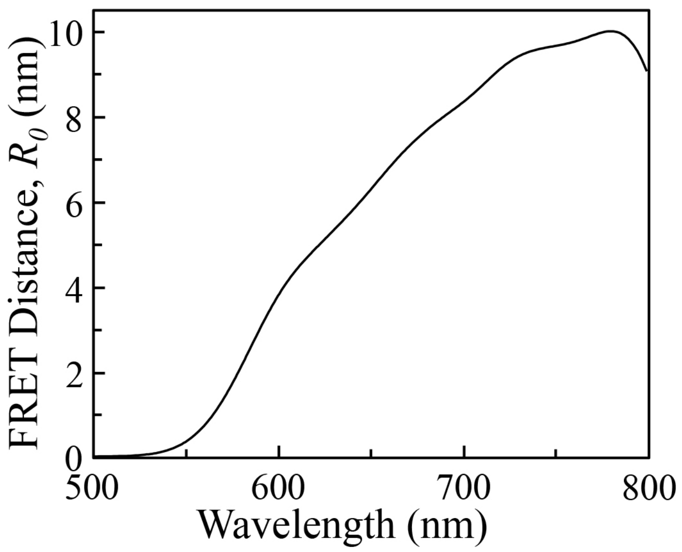

Supplementary Figure 14 | Dependence of FRET distance on excitonic wavelength of CdSe in ND-CdSe. This theoretical curve shows clear dependence of FRET distance on excitonic energy of semiconductor quantum dots (CdSe), which can be experimentally tuned by tailoring the size of quantum dots due to the quantum confinement effect.

SiO_2 shell thickness & corresponding TEM images in main text	Volume of Ethanol (in 4% ammonia water)	TEOS		Repeated growth process
		Volume	Volume ratio in ethanol	
14.8 nm (Fig.4a)	10 ml	250 μL	1%	None
3.0 nm (Fig.4b)	10 ml	15 μL	1%	None
7.5 nm (Fig.4c)	10 ml	30 μL	1%	None
12.2 nm (Fig.4d)	10 ml	125 μL	1%	None
35.3 nm (Fig.4e)	10 ml	100 μL	1%	Four times
90.1 nm (Fig.4f)	10 ml	25 μL	10%	Five times

Supplementary Table 1 | Summary of synthetic conditions for different SiO_2 shell thickness in ND-SiO_2. Typical samples achieved by following growth conditions in table are presented in Figures 4a-4g in the main text and Supplementary Fig. 9.

Supplementary Note 1: Sample Homogeneity of Hybrid ND Based Nanostructures

We have evaluated our sample quality by statistical analysis of extensive TEM images of nanostructures.

We have observed that morphology of pure NDs as purchased from Adamas Nanotechnologies Inc shows a plate-like shape (see Supplementary Figs. 1a and 1b). This might be due to the preparation method of NDs by ball milling, in which larger size diamonds break preferably along (111) plane (for example, Supplementary Fig. 1c). As a result, the overall shape of pure NDs shows certain irregularity and polydispersity.

The average size and size distribution of nanoparticles on the surface of NDs in one batch of hybrid nanostructures (see Figs. 2a-2f and Supplementary Fig. 2) are obtained by counting about 50 hybrid nanostructures. The surface coverage of nanoparticles in one hybrid nanostructure is defined as the total number of nanoparticles on the surface divided by two times of apparent surface area of ND measured by TEM images. The coefficient of two here is due to the bottom and top surfaces of the ND. The average coverage and its distribution in one batch of sample (see Figs. 2g-2l and Supplementary Fig. 2) are obtained by counting more than 70 hybrid nanostructures. The uncertainties/error bars of both size and surface coverage density are determined by the standard deviation in a statistical analysis.

We would like to further comment on our evaluation of the size and shape distributions in as-synthesized hybrid nanostructures. Firstly, our estimation of size distribution in Supplementary Fig. 2 is likely overestimated. The ND is a three-dimensional plate- like

structures, and TEM is a two-dimensional projection imaging technique. As a result, the nanoparticles with same size in the bottom and on the top surfaces of a ND manifest different "apparent" size (with slightly different contrast) in the same image due to different focusing plane. Supplementary Fig. 4 presents one selected ND surface with grown Ag nanoparticles under the condition for high surface coverage, and the red and yellow dashed circles mark nanoparticles on the top and bottom surfaces, respectively. It is worth noting that this scenario is different from pure spherical nanoparticles in which all nanoparticles can be imaged in the same focusing plane. This fact makes estimation of size inhomogeneity more difficult for ND based hybrid nanostructures. However, in order to avoid subjective judgement in our data analysis, we have decided to evaluate size variation based on their apparent size in high-resolution TEM images without correcting for any focusing effects. As a result, we believe our estimation of size inhomogeneity is over-estimated. Secondly, under medium and high surface coverage we have found that some neighboring metal nanoparticles on the ND's surface touch with each other due to growth in close proximity. This can also be clearly seen in Supplementary Fig. 4b. To confirm this observation, we have also performed simulations by randomly distributing Ag nanoparticles on a ND surface under different surface coverage and compared three different coverage densities in Supplementary Figs. 4c-4e. This simulation clearly shows an increasing tendency for touching nanoparticles under medium and high coverage (see examples highlighted by red arrows), which agree well with our TEM observation in Supplementary Fig. 4b. One immediate effect is that under medium or high surface coverage the shape and size of metal nanoparticles can appear irregular, particularly in the low-resolution images. Lastly, the overall shape irregularity of hybrid nanostructures

originates from the starting commercial NDs, as discussed above. To account for this effect, when we perform statistical analysis of surface coverage, we have measured the surface coverage density as defined above instead of simply counting the number of nanoparticles on the surface per ND. Given the irregularity and polydispersity of commercial ND source, uniformity of our surface coverage density summarized in Supplementary Fig. 2 is good. More importantly, our achieved control of these important structural parameters can allow us to study their dependent optical properties. Clear coupling between the NV and plasmon has been observed with correct tendency, as summarized in Fig. 6 in the main text.

Supplementary Note 2: Comparing Hybrid ND-Ag Nanostructures with Mixtures of Ag Nanoparticles and NDs

In order to exclude the possibility that our as-synthesized hybrid nanostructures are due to mixture of Ag nanoparticles and NDs, we have prepared and characterized samples by mixing Ag nanoparticles with NDs with different ratio, and presented results in Supplementary Fig. 5. By comparing mixtures with as-synthesized hybrid nanostructures, we can safely conclude that our ND-Ag hybrid nanostructures is not a mixture product of NDs and Ag nanoparticles based on the following three observations: First, in the mixture samples, most of the Ag nanoparticles aggregate in the areas without NDs. By contrast, all of our as-grown ND based hybrid nanostructures are clean without the aggregation of freestanding nanoparticles; Second, in the mixture samples, even though occasional Ag nanoparticles can be found to stay on top of ND surfaces, majority of NDs are clean with an absence of Ag nanoparticles. This is different from our observation of uniform coverage of Ag nanoparticles in the as-synthesized hybrid nanostructures; and lastly, we have compared samples with different mixture ratio of Ag nanoparticles to NDs, and we have not observed big difference among different mixture ratios except that more aggregated freestanding Ag nanoparticles can be found in the areas without NDs for the mixtures with higher concentration of Ag nanoparticles. On the other hand, surface coverage of Ag nanoparticles in as-synthesized hybrid nanostructures can be well controlled by varying synthetic condition (see also Figs. 2g-2l in the main text and Supplementary Fig. 3).

According to this control experiment, we can safely exclude the possibility of the mixture of Ag nanoparticles and NDs in our hybrid samples.

Supplementary Note 3: Suppression of Fluorescence of Metal Oxide in a Hybrid ND-Metal Nanostructure

In our experiment, we have noticed that photoionization of metal oxide formed on the surface of metal nanoparticles can give very bright and unstable fluorescence[1]. Such unwanted fluorescence can be detrimental to the optical measurement of NV centers, if no sufficient care is taken. We have addressed this surface oxide issue by preparing our sample under nitrogen protection and sealing the sample with a polymer (PMMA). Our method is sufficient to suppress the formation of metal oxide on the surface of metal nanoparticles (thus its related fluorescence). To confirm this we have compared fluorescence of samples with and without polymer protection, and presented results in Supplementary Fig. 12. These measurements were performed using a 532 nm CW laser as the excitation source and the fluorescence was collected through a 600 nm long-pass filter using a QE65000 spectrometer from Ocean Optics. The spectra difference with and without polymer protection is dramatic. The spectrum acquired from protected sample shows similar feature to that of pure ND, confirming the absence of the Ag oxide related fluorescence. In addition, the fluorescence of surface silver oxide is typically bright compared to the NV center and shows widely fluctuating intensity over time (Supplementary Fig. 12b).

Supplementary Note 4: Ultrafast Fluorescence Lifetime Measurement and Data Fitting

For fluorescence lifetime measurements, the illumination laser pulse is selected by using a 10 nm bandwidth filter centered around 532 nm from femtosecond supercontinuum white light beam that is generated by focusing a pulsed Ti:S laser (Spectra Physics, Tsunami) into a photonic crystal fiber. Fluorescence is collected with an APD and lifetime curves are collected using a time correlated single photon counting card (Becker & Hickl). The repetition rate of the Ti:S laser is 80 MHz. As a result, fluorescence decay is typically collected in range of 12.5 ns. In addition, we have intentionally avoided the first nanosecond of collected lifetime data by fast temporal gating. This is necessary in order to further suppress the contribution of intrinsic metal fluorescence, which will be discussed below.

Lifetime curves are fit with a bi-exponential curve, $I=\sum_{n=0}^{2}\left[Ae^{\left(-\frac{t+n\bullet C}{\tau_1}\right)}+Be^{\left(-\frac{t+n\bullet C}{\tau_2}\right)}\right]$, where I represents counts, A, B, τ_1 and τ_2 are fitting parameters, and C is the constant of 12.5 ns to account for any incomplete fluorescence decay from previous excitation pulses. The lifetime τ_1 is short and associated with the instrument response function of the APD. The fit also ignores the first nanosecond of the fluorescence decay in order to eliminate any contributions from fluorescence generated by the metal nanoparticles (even though intrinsic photoluminescence of metal nanoparticles is extremely small). Other than the unwanted fluorescence from metal oxide that we have discussed in Supplementary Note 3, it has been shown that metal nanoparticles have their own intrinsic fluorescence

originating from inter- and intra-band transitions with a lifetime on the order of picoseconds[2]. By ignoring the first nanosecond of decay data, contribution from metal nanoparticles to fluorescence lifetime measurement of NV centers in a hybrid nanostructure can thus be minimized. The first lifetime, τ_1, is typically ~1.1 ns and is a result of the mono-exponential tail of the APD instrument response function caused by diffusion in the detector. While fast temporal gating removes the majority the lifetime effects from the metal fluorescence, it is necessary to account for longer-lived detector effects using our fitting method. Data is collected from more than 100 hybrid nanostructures and binned to a histogram for each sample. The main cause of the distribution of lifetime with in each sample is attributed to variation in the orientation of the NV center dipole[3]. A typical fluorescence lifetime trace and corresponding fitting is provided in Supplementary Fig. 13 with following fitting parameters: A=37.277, B=19.277, τ_1=1.1 ns and τ_2= 6.82 ns.

We also would like to point out that a higher fluorescence rate of NV centers by plasmonic coupling does not necessarily mean brighter emission. In order to determine the radiative and non-radiative enhancement it will require further experimentation. For example, it needs to compare in-situ emission intensity before and after growth of external nanoparticles, but in our current experiment we have directly synthesized freestanding ND-metal nanoparticles hybrid nanostructures, so we cannot perform such in-situ measurement. Alternatively, if the quality of commercial ND source can be improved in the future to contain a uniform number of NV centers, the evaluation of radiative and non-radiative enhancement could also be feasible.

Supplementary Methods

All chemicals were used as received without further processing. Acetone (99%), Ammonium hydroxide (NH_4OH, 28.0-30.0%), Cadmium nitride tetrahydrate ($Cd(NO_3)_2 \bullet 4H_2O$, 99.999%), Ethanol (anhydrous, 99.5+%), Gold(III) chloride trihydrate ($HAuCl_4 \bullet 3H_2O$, 99.9+%), Sodium borohydride ($NaBH_4$, 99.99%), Sodium hydroxide (NaOH, 99.99%), Polyvinylpyrrodlidone (PVP, average M_w~55,000), Silver nitrate ($AgNO_3$, 99+%), Selenium (Se, powder, -100 mesh, 99.5+%), Toluene (anhydrous, 99.8%), Tetraethyl orthosilicate ($Si(OC_2H_5)_4$, TEOS), Tributylphosphine (TBP, 97%) were purchased from Sigma-Aldrich. Tin(II) chloride ($SnCl_2$, 98+%) was purchased from Acros. Nanodiamonds (part #: ND-(1-4)NV-40nm) were purchased from Adamas Nanotechnologies, Inc. These NDs are ball-milled type *IIb* diamonds with size of 40nm and 1-4 NV centers per nanodiamond on average. Characterization of pure NDs after cleaning is presented in Supplementary Fig. 1 with detailed discussion in Supplementary Note 1.

The fresh NaHSe solution is prepared by following the recipe below as the Se precursor utilized in current study: ice-cold 50 ml of water is purged with dry nitrogen gas for 30 mins. 0.037 g of Se powder is kept in an ice-cold glass vial with addition of a few drops of acetone, followed by drying with nitrogen gas flow. 0.060 mg of $NaBH_4$ and 1.0 ml of nitrogen protected water are added to the glass vial containing Se powder, and the mixture is sealed with parafilm immediately and kept in ice-water bath for 30mins until the black powder is turned to colorless, which indicates formation of concentrated NaHSe solution.

Deionized water (H_2O) was obtained by using Barnstead NANOPure water purification system, having a resistivity of 18.3 MΩ-cm. All aqueous solutions were prepared in the deionized water.

Supplementary References

1. Peyser, L. A., Vinson, A. E., Bartko, A. P. & Dickson, R. M. Photoactivated fluorescence from individual silver nanoclusters. *Science* **291**, 103-106 (2001).

2. Dulkeith, E. *et al.* Plasmon emission in photoexcited gold nanoparticles. *Phys.Rev. B***70**, 205424 (2004).

3. Tisler, J. *et al.* Fluorescence and spin properties of defects in single digit nanodiamonds. *ACS Nano* **3**, 1959-1965 (2009).
